# Can integrated care improve the efficiency of hospitals? Research based on 200 Hospitals in China

**DOI:** 10.1186/s12962-021-00314-3

**Published:** 2021-09-22

**Authors:** Zixuan Peng, Li Zhu, Guangsheng Wan, Peter C. Coyte

**Affiliations:** 1 Institute of Health Policy, Management and Evaluation, Dalla Lana School of Public Health, University of Toronto, Toronto, Canada; 2 School of Political Science and Public Administration, Guangxi University for Nationalities, Nanning, China; 3 School of Nursing & Health Management, Shanghai University of Medicine & Health Sciences, Shanghai, China

**Keywords:** Integrated healthcare, Hospital efficiency, DEA methods

## Abstract

**Background:**

The shift towards integrated care (IC) represents a global trend towards more comprehensive and coordinated systems of care, particularly for vulnerable populations, such as the elderly. When health systems face fiscal constraints, integrated care has been advanced as a potential solution by simultaneously improving health service effectiveness and efficiency. This paper addresses the latter. There are three study objectives: first, to compare efficiency differences between IC and non-IC hospitals in China; second, to examine variations in efficiency among different types of IC hospitals; and finally, to explore whether the implementation of IC impacts hospital efficiency.

**Methods:**

This study uses Data Envelopment Analysis (DEA) to calculate efficiency scores among a sample of 200 hospitals in H Province, China. Tobit regression analysis was performed to explore the influence of IC implementation on hospital efficiency scores after adjustment for potential confounding. Moreover, the association between various input and output variables and the implementation of IC was investigated using regression techniques.

**Results:**

The study has four principal findings: first, IC hospitals, on average, are shown to be more efficient than non-IC hospitals after adjustment for covariates. Holding output constant, IC hospitals are shown to reduce their current input mix by 12% and 4% to achieve optimal efficiency under constant and variable returns-to-scale, respectively, while non-IC hospitals have to reduce their input mix by 26 and 20% to achieve the same level of efficiency; second, with respect to the efficiency of each type of IC, we show that higher efficiency scores are achieved by administrative and virtual IC models over a contractual IC model; third, we demonstrate that IC influences hospitals efficiency by impacting various input and output variables, such as length of stay, inpatient admissions, and staffing; fourth, while bed density per nurse was positively associated with hospital efficiency, the opposite was shown for bed density per physician.

**Conclusions:**

IC has the potential to promote hospital efficiency by influencing an array of input and output variables. Policies designed to facilitate the implementation of IC in hospitals need to be cognizant of the complex way IC impacts hospital efficiency.

**Supplementary Information:**

The online version contains supplementary material available at 10.1186/s12962-021-00314-3.

## Background

**Fig. 1:**
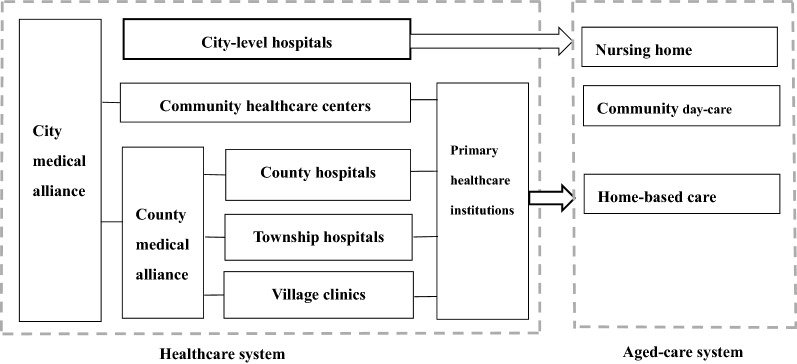
Integration of health-care institutions in China. The author visualized the structure of IC in China based on a policy review. IC in China includes: (1) Vertical integration among different types of healthcare institutions or aged-care institutions; (2) Horizontal integration among the healthcare institutions and aged-care institutions

China, like many other countries, is facing both a greying of the population and an increased prevalence of chronic, non-communicable diseases. Those over 65 years of age represented 11.9% of the population in 2018 but are expected to account for 20% by 2040 [1, 2]. Likewise, the prevalence of chronic, non-communicable disease (NCD) among those over 65 years of age was 65% in 2008 and increased to 75% by 2018 [3, 4]. Older people with chronic diseases usually suffer from problems in the physical, psychological and social domains [5], and have diverse and complex needs in the areas of prevention, treatment, etc. [6]. As people age, the risk of chronic conditions increases, and this is estimated to increase the national burden of NCDs in China to 40% by 2030 [7]. Under the twin pressures of ageing and a high prevalence of chronic diseases, integrated care has been proposed as a potential solution for China. IC encompasses various methods of funding, organization and delivery of care to enhance system efficiency [6, 8–10]. Health systems realize their goals at all levels through enhanced hospital performance [11]. This is especially the case in China where hospitals may benefit most from IC through the provision of comprehensive and coordinated care. As shown in Fig. 1, Chinese hospitals cooperate with other institutions to achieve vertical and horizontal integration [12].

Efficiency studies contribute to informed decision-making as the findings from such studies may identify opportunities to improve care performance in hospitals and at the same time contain resource consumption [13]. However, studies have seldom looked at the impact of IC on hospital efficiency. Most studies have focused on measuring health outcomes among the elderly that may be attributed to the implementation of IC [14–24]. Furthermore, it remains unclear from that literature the direction of effect, if any, of IC on hospital efficiency. Some studies demonstrated that integrated partnerships and a coordinated continuum of services dedicated to the treatment of specialized diseases or a defined population may improve hospital efficiency [25–29]. However, weak and, on occasions, negative impacts of IC on hospital efficiency were also found [30, 31]. As such, there is an opportunity to add to the literature by directly assessing the impact of IC on hospital efficiency.

The purpose of this paper is three-fold: first, to investigate potential differences in efficiency between IC hospitals and non-IC hospitals; second, to examine variations in efficiency among different types of IC hospitals; and third, to explore whether the implementation of IC impacts hospitals efficiency. The paper is structured in the following manner: In “Methods” section, we explain data sources, variables and the methods of analysis. The results are outlined in “Results” section and discussed in “Discussion” section. We end with a brief conclusion that highlights several policy implications.

## Methods

### Data sources

Our study chose C city as the sample for three reasons: first, C city is among the first batch of cities to implement IC in China. According to the “Notice Regarding the Determination of the First Batch of National-level Pilot Cities of Integrated Care” [32], C city is among the first of two cities to implement IC in H province. Pilot cities provide financial and administrative support and hospitals participated on a voluntary basis. Second, C city is in central China and is representative of all China in having average economic and social development. Finally, the city was selected for reasons of data accessibility. Specifically, the data were obtained directly from the Provincial Bureau of Statistics that links a wide range of administrative databases to hospital-level data. We used a dataset which was formally collected by the Provincial Bureau of Statistics in 2016 and all the hospitals in C city reported their data according to the requirements of the government. To ensure maximum representativeness, all hospitals in C city were included in our research. The dataset used contains information on personnel, equipment, cost and revenue data for each of 200 hospitals in C city, H province in 2016.

### Study variables

Hospital efficiency was the outcome of our study. In economic theory, average productivity is calculated as a ratio of outputs to inputs. Applications of efficiency measurement have extended this concept by using these ratios to construct “best practice” frontiers. In most cases, inputs to the production function of health services include capital (e.g., medical equipment, hospital beds, etc.), labor (e.g., human resources), land and raw materials. Outputs include health services provided (e.g., number of surgeries performed) [33]. Guided by our literature review on efficiency analysis [13, 34–39], we included as many input and output variables as possible. Specifically, Output variables included length of stay, inpatient admissions, outpatient visits, emergency visits, family visits, revenues, number of surgeries, and number of discharges from hospital. Input variables comprised operating cost, number of physicians, number of ancillary medical staff, number of nurses, number of other staff (including administrative, technical and logistic staff) as well as number of hospital beds.

In 2016, C city started to implement IC policy and hospitals could voluntarily decide whether to participate. Our research included the implementation of IC as a dummy independent variable and tests to see if it was positively association with hospital efficiency [29]. Additional control variables were also considered in our analysis. The increasing complexity of healthcare and resulting clinical specialization may result in the fragmentation of healthcare, thereby compromising patient safety and hospital efficiency [40]. In our research, we used the number of key clinical departments as a proxy for clinical specialization and we expected that it would be negatively correlated with hospital efficiency. Moreover, facility type was also found to be a useful predictor of hospital efficiency whereby facilities operating at a large scale may realize greater technical efficiency due to increasing returns to scale [30]. Third, a higher mortality rate (low quality health services) was found to raise the costs of the hospitals [34] and thereby to erode hospital efficiency. Fourth, shorter average length of stay was expected to improve the use of medical beds and enhance efficiency [41]. Fifth, we also included bed density per physician and bed density per nurse as control variables, because we expected these variables to be positively associated with hospital efficiency [13].

### Statistical analysis

#### Data envelope analysis method

Non-parametric Data Envelopment Analysis and parametric Stochastic Frontier Analysis are the two main approaches to the measurement of efficiency. We employed DEA because of its ease of implementation, its nonparametric basis and substantial freedom on the specification of inputs and outputs [42]. As shown in Eq. (1), the efficiency score *θ* for a hospital* i* is measured relative to the efficiency of the other hospitals (*i* = *1,…,n*), subject to the restriction that all hospitals are on or below the efficient production frontier [43]. The value of each hospital’s measure of efficiency ranges from 0 to 1. Efficient hospitals are those on the efficient frontier and their efficiency score is 1, while inefficient hospitals lie below the efficiency frontier and their efficiency score is less than 1. The further theses inefficient hospitals are away from the efficiency frontier, the lower is their efficiency score. In this paper, we adopted an input-oriented DEA model that focuses on minimizing the use of inputs in order to produce a given output [13]. Furthermore, variable returns to scale (VRS) was considered by our study based on two considerations: (1) in most cases, hospitals have varying sizes and this is factor that determines their efficiency [44]; and (2) public hospitals in China were not only natural monopolies but also administrative monopolies [45]. To investigate the efficiency differences among different types of IC, IC was classified into contractual, administrative, insurance-driven and virtual integration by our previously published study [46]. The contractual, administrative and virtual integration types were found in our research. The definition, core strategy, strengths and weaknesses of each IC type were summarized in Table 1.

1 $$\hat{\psi }_{DEA} = \{ (x,y) \in R_{ + }^{p + q} |y \le \sum\limits_{i = 1}^{n} {\theta_{i} Y_{i} ,\,x} \ge \sum\limits_{i = 1}^{n} {\theta_{i} X_{i} ,\,for\;(\theta_{1} , \cdots \theta_{n} )\;S.t.\sum\limits_{i = 1}^{n} {\theta_{i} = 1;\;\theta_{i} \ge 0,\,i = 1, \cdots ,n\} } }$$

**Table 1:** Different types of IC in China

Type	Definition	Strategy	Strengths	Weaknesses
Contractual integration (CI)	CI seeks to build cooperative relationships among different institutions through formal contracts	Contract	*Flexible*: Healthcare institutions are flexible to cooperate in specific areas; *Trustful*: Formal cooperative relationships could be formed between and among member institutions	*Insufficient:* Contracts can only cover certain areas of IC and is not sufficient to ensure the thorough and effective implementation of IC
Administrative integration (AI)	AI is featured with administrative characteristics that newly-built councils conduct united but limited management over financial, personnel, and property resources within the IC network	Management	*Equal*: Governments implement united but limited management over resources and therefore the distribution of resources could be more equal; *Powerful:* AI is usually led by the officials of the government and therefore is powerful in implementing IC under the context of the Chinese political system	*Incentive-lacking:* Resource-rich public hospitals are unwilling to support primary healthcare institutions who need help; Private healthcare institutions lack incentives to participate due to their interest-seeking behaviour patterns
Insurance-driven integration (II)	II is mainly adopted by institutions covered by the same type of medical insurance	Insurance	*Consistent:* Member institutions are less likely to encounter barriers caused by different funding polices when implementing IC; People could be referred to different institutions under the same reimbursement policy	*Geographically limited:* It is difficult for institutions that are located in different administrative regions to cooperate
Virtual integration (VI)	VI is an emerging form of IC, with its emphasis on making full use of modern information technology	Technology	*Accessible:* It is beneficial for institutions located in remote rural areas to cooperate with healthcare institutions in developed areas; *Resource-saving:* Since services are provided via technological devices, patients could save accommodation and transportation expenditures	*Inconsistent:* Healthcare institutions can only receive virtual support, which is limited in the long run; Patients cannot receive continuous healthcare services and they still need to visit hospitals in person after receiving online virtual diagnosis

The authors compiled the table based on a previous published paper and a policy review

#### Tobit regression

The efficiency score is the outcome of interest. This dependent variable is limited in its range with values that lie within the unit interval, i.e., between 0 and 1. To ease interpretation, the efficiency scores were transformed to represent inefficiency scores using the transformation in Eq. (2) [13]. After transformation, the inefficiency score for efficient hospitals is 0, while inefficient hospitals have inefficiency scores that exceed 0. Given the value of the dependent variable is censored at zero, Tobit regression was used in our study. In our research, inefficiency is measured by a set of input and output variables. To further explore how IC influences the inefficiency score through these input/output variables, we regressed each input and output variable on the dummy IC variable.

2 $$Inefficiency\;score = \left( {\frac{1}{Efficiency\;score} - 1} \right)$$

#### Propensity score matching

The causal effects of IC on hospitals efficiency cannot be estimated using ordinary regression due to potential selection bias associated with confounding variables. Propensity score matching (PSM) was used to reduce such potential bias associated with confounding variables in the decision to implement IC, and PSM is useful to identify potential causal effects of IC on hospital efficiency. Following the analytical process of Staffa [47], Garrido [48], Caliendo [49] and Austin [50], we performed PSM in three steps: first, we calculated the probability of implementing IC given the observed covariates using logistic regression analysis. The covariates included were those that were expected to be related to both the implementation of IC and were expected to be important determinants of hospital inefficiency [48]. These variables included hospital type, inpatient mortality rate, hospital capacity, average length of stay for discharged patients, bed density per physician, and bed density per nurse. Second, we employed the K-nearest neighbor matching method with a matching ration 1:1 and a caliper value of 20% of the standard deviation of the logit of the estimated propensity score [51]. Finally, balance diagnostics of the matching results were undertaken through use of a chi-square test (for categorical variables) and two sample t-test (for continuous variables). We set 0.20 as the threshold of the required standard deviation, given the size of the sample used in our study [48, 52–54].

#### Sensitivity analyses

To check the robustness of our research results, we conducted the following sensitivity analyses: first, we conducted direct ordinary least squares regression analysis to investigate the difference associated with different estimation methods; second, we performed Tobit regression using all the sample hospitals. This allowed us to compare the results with PSM and without PSM; third, we used constant returns to scale (CRS) to provide comparisons and test for stability, variability and robustness of efficiency results obtained using the VRS. All analyses mentioned above were conducted using R [55].

## Results

### Descriptive results

Table 2 describes the characteristics of the sample of hospitals in this study. There were 24 IC hospitals (12%) in 2016. About 23.5% of hospitals (N = 47) were regional medical centers. The number of key clinical departments recognized by the government varied from 0 to 31 with a mean and SD of 2.05 and 3.97, respectively. The number of key clinical departments in IC hospitals (mean = 6.00; SD = 8.06) was substantially larger than those in non-IC hospitals (mean = 1.52; SD = 2.64). The average length of stay for discharged patients in IC hospitals was 23.83 days (SD = 43.44), which was substantially larger than that in non-IC hospitals (mean = 10.40; SD = 12.20) and in all the 200 hospitals (mean = 12.02; SD = 19.19). Overall, the mean inpatient mortality rate was small at 0.23% with SD of 0.01 and was smaller among IC hospitals than that in non-IC hospitals (P < 0.001). Bed density per physician and bed density per nurse averaged at 5.09 (SD = 5.31) and 3.68 (SD = 3.90), respectively, with no significant difference found between IC and non-IC hospitals.

**Table 2:** Descriptive statistics for the sample hospitals

Code	Explanation of the variable	N	Mean	SD	Median	Min	Max
Input variable							
NP	Number of physicians	200	86.593	175.949	20	1	1265
NAMS	Number of ancillary medical Staff	200	3.722	3.953	3	0	30
NN	Number of nurses	200	144.742	338.667	33	0	2684
NOE	Number of other employeesstaff, including administrative, technical staff and logistic staff	200	111.792	176.389	54	2	1,563
NB	Number of hospital beds	200	303.970	556.952	93	0	4042
OO	Operating cost	200	192,476.615	599,029.829	22,317	854	5,283,269
Output variable							
ND	NAnnual number of discharges from hospital	200	9835.319	20,289.562	2688	0	136,788
UD	Length of stay (bed days per year)	200	100,289.537	205,961.229	20,471	0	1,439,541
NIA	Annual nNumber of inpatient admissions	200	9842.152	20,305.706	2665	0	136,926
NOV	Annual nNumber of outpatient visits	200	119,606.523	350,487.324	14,999	0	2,870,064
NEV	Annual nNumber of emergency visits	200	20,926.295	43,641.697	6480	0	396,063
AVFP	Annual nNumber of annualfamily visits for family planning	200	1019.411	3916.575	1019	0	53,515
ARH	Annual revenues of hospitals	200	179,652.965	596,434.739	15,757	108	5,179,985
NS	Annual nNumber of surgeries	200	3597.900	8437.977	2527	0	71,788
Independent variable							
IC1	Whether implementing IC or not	200	Yes: n = 24 (12%); No: n = 176 (88%)				
ROPA	Average length of stay for discharged	200	12.016	19.188	9	1	193
NAPP	Beds density per physician	200	5.092	5.314	4	0	40
NAPN	Bed density per nurse	200	3.683	3.896	3	0	31
RMA	Inpatient mortality rate	200	0.002	0.008	0	0	0
WHC	Facility type measure by whether the hospital is a regional medical center or not	200	Yes: n = 47 (23.5%); No: n = 152 (76%)				
TNS	Clinical specialization measured by the number of key clinical department	200	2.058	3.971	2	0	31
Dependent variable							
INEFF(VRS)	Inefficiency score of hospital	200	0.346	0.478	0	0	3
INEFF(CSR)	Inefficiency score of hospital	200	0.512	0.836	0	0	8

### Efficiency of hospitals

Table 3 reports the average efficiency scores of hospitals. Most hospitals obtained efficient scores, i.e., they were on the efficient production frontier. The mean efficiency score for hospitals was 0.81 when the VRS was used. A large percentage of these hospitals (N = 83, 41.5%) operated at their optimal level. Furthermore, 17% of hospitals (N = 37) had efficiency scores ranging from 0.7 to 0.9, here classified as being moderately efficient. Only 4 hospitals had an efficiency score of less than 0.4, here classified as being extremely inefficient. When the efficiency scores were estimated using the CRS, the mean efficiency score fell to 0.76. In this CRS model, over 60 hospitals (31%) were identified as being efficient. Compared to the VRS model, fewer hospitals under the CRS model were efficient and the number of hospitals identified as moderately efficient (N = 50, 25%) and extremely inefficient (N = 9, 4.5%) also increased.

**Table 3:** Average efficiency scores of hospitals

Hospital	Efficiency score (VRS)	Efficiency score (CRS)	Scale efficiency score
Mean efficiency score of IC hospitals	0.957	0.875	0.916
Mean efficiency score of AI	1	1	1
Mean efficiency score of CI	0.949	0.850	0.900
Mean efficiency score of VI	1	1	1
Mean efficiency score of non-IC hospitals	0.790	0.739	0.765
Mean efficiency score of all hospitals	0.810	0.755	0.783

IC hospitals were expected to operate more efficiently than their non-IC counterparts. The mean CRS and VRS efficiency scores for IC hospitals was 0.88 and 0.96, respectively, which on average was larger than the scores for non-IC hospitals (0.74 and 0.80 respectively). These differences were statistically significant (P = 0.004 in the CRS model; P < 0.001 in the VRS model). The scale efficiency score, which is the mean of the CRS and VRS efficiency scores [13], was 0.92 for IC hospitals and substantially larger (P = 0.001) than that for non-IC hospitals (0.77). Meanwhile, the efficiency scores of the three different IC types were also reported in Table 3. It was found that virtual and administrative integration, on average, obtained higher efficiency scores than contractual integration.

### The influence of IC on efficiency

Our research found out that the potential bias caused by confounding covariates was eliminated after matching. Adequate overlap between the IC hospitals and the non-IC hospitals was shown in Additional file 1: Figure S1, and this implies that we could perform PSM using our dataset. Moreover, the results of the chi-square test and the Welch Two Sample t-test were shown in Additional file 1: Table S1. After matching, no statistically significant difference in covariates were found between IC hospitals and non-IC hospitals. The mean of the difference in covariates between IC hospitals and non-IC hospitals was balanced after matching. No covariate had an absolute standard difference of more than 20% after matching and the mean standardized difference dropped from 42.62 to 13.71% (Additional file 1: Table S2 and Figure S3).

Table 4 reports the Tobit regression results. Non-IC hospitals were expected to achieve higher inefficiency scores than IC hospitals. In model 1, the estimated coefficient of IC was -0.59 with a 95% CI between − 0.01 and 0.17. When adjusting for all the covariates (model 2), the coefficient of IC was slightly smaller at -0.54 with a 95% CI between − 0.85 and − 0.23. This implies that compared with IC hospitals, non-IC hospitals were expected to achieve 0.54 higher inefficiency score. This model also identified that bed density per nurse was a positive predictor of higher inefficiency. In contrast, the inefficiency score of hospitals that were regional medical centers was found to be 0.34 lower than other hospitals. Similarly, the number of key clinical departments and the bed density per physician were found to be negatively associated with inefficiency scores. Meanwhile, the results of the CRS model only presented slight differences compared with the VRS model.

**Table 4:** The impact of different factors on the inefficiency score of hospitals using Tobit regression

	Model 1				Model 2			
	Estimate (Std.Error)	t-value	Pr( >|t|)	95%CI	Estimate (Std.Error)	t-value	Pr( >|t|)	95%CI
Intercept	0.218 (0.145)	1.499	0.134	[− 0.067, 0.502]	0.485 (0.153)	3.174	0.002**	[0.186, 0.785]
IC1	− 0.592 (0.215)	− 2.756	0.006**	[− 1.012, − 0.171]	− 0.538 (0.159)	− 3.390	0.001***	[− 0.848, − 0.227]
RMA					− 19.490 (12.388)	− 1.573	0.116	[− 43.769, 4.790]
WHC					− 0.337 (0.162)	− 2.087	0.037*	[− 0.654, − 0.020]
TNS					− 0.054 (0.018)	− 3.065	0.002**	[− 0.088, − 0.019]
NAPP					− 0.169 (0.056)	− 3.026	0.003**	[− 0.278, − 0.060]
ROPA					0.003 (0.004)	0.744	0.457	[− 0.005, 0.010]
NAPN					0.354 (0.097)	3.658	0.000***	[0.165, 0.544]
Variance of model	− 0.47329 (0.1699)	− 2.783	0.005**	[− 0.806, − 0.140]	− 0.935 (0.162)	− 5.764	0.000 ***	[− 1.252, − 0.617]

Significance codes: ‘***’ ≤ 0.001; ‘**’ ≤ 0.01; ‘*’ ≤ 0.05

The influence of IC on each input and output variable was reported in Table 5. IC was expected to be associated with a set of input and output variables. The number of physicians, nurses, other employees, and beds in IC hospitals were significantly larger than those in the non-IC hospitals. The same positive influence of IC on discharges, length of stay, inpatient visits, and emergency visits was found. The goodness of fit (R^2^) was generally low at 10% for input variables and 8% for output variables. The P-value for the F-test for all the models was smaller than 0.05, implying that all the models passed the joint hypothesis test.

**Table 5:** The influence of IC on output and input variables

Dependent variable	Estimate (Std.Error)	t-value	Pr( >|t|)	95%CI
Output variable				
ND	19,179 (8,766)	2.188	0.034*	[1,511.749, 36,845.56]
UD	229,088 (86,403)	2.651	0.011*	[54,955.40, 403,221.4]
NIA	19,246 (8,763)	2.196	0.033*	[1,584.254, 36,906.88]
NOV	237,400 (153,958)	1.542	0.130	[− 72,882.971, 547,682.4]
NEV	53,604 (21,171)	2.532	0.015*	[10,936.58, 96,271.92]
AVFP	− 2,470 (2,300)	− 1.074	0.289	[− 7,105.227, 2,164.915]
ARH	361,001 (258,554)	1.396	0.170	[− 160,081.04, 882,083.0]
NS	6,432 (3,689)	1.743	0.088	[− 1,003.135, 13,866.44]
Input variable				
NP	165.70 (71.34)	2.322	0.025*	[21.912, 309.480]
NAMS	− 1.2416 (1.217)	− 1.020	0.313	[− 3.694, 1.211]
NN	344.4 (141.4)	2.435	0.019*	[59.401, 629.469]
NOE	129.34 (64.04)	2.020	0.050*	[0.265, 258.412]
NB	649.3 (236.4)	2.747	0.009**	[172.932, 1,125.676]
OO	334,981 (262,901)	1.274	0.209	[− 194,861.68, 864,823.9]

Significance codes: ‘***’ ≤ 0.001; ‘**’ ≤ 0.01; ‘*’ ≤ 0.05

### Results of sensitivity analyses

The results of sensitivity analyses were reported in Table 6. We first conducted ordinary least square regression analysis. It was demonstrated that in the VRS model, the coefficient on IC for hospital inefficiency was − 0.35, which was smaller than the results derived from the Tobit regression. When adjusting for all the covariates, the coefficient on IC was − 0.33, which was also smaller than that in the model where Tobit regression was performed. Second, we compared the results with PSM and without PSM. Compared with models using PSM, the same negative, but larger, influence of IC on hospital inefficiency (coefficient was − 0.65) was found in the VRS model without PSM. When adjusting for all the covariates, the negative influence of IC on hospital inefficiency (coefficient was − 0.43) was still found in the VRS model. Moreover, under the CRS assumption, the positive influence of IC implementation on hospitals efficiency was found to be smaller at − 0.433 and − 0.423 for the CRS model without and with covariates, respectively. These results imply that our research results were robust to these considerations.

**Table 6:** Results of sensitivity analyses

	OLS				Without PSM				VRS			
	Estimate (Std.Error)	t-value	Pr( >|t|)	95%CI	Estimate (Std.Error)	t-value	Pr( >|t|)	95%CI	Estimate (Std.Error)	t-value	Pr( >|t|)	95%CI
Intercept	0.403(0.076)	5.284	0.000***	[0.250,0.556]	0.201(0.058)	3.449	0.001***	[0.087,0.314]	0.422(0.121)	3.483	0.000***	[0.184,0.658]
IC1	− 0.346(0.108)	− 3.213	0.002**	[− 0.564,− 0.129]	− 0.647(0.186)	− 3.485	0.000***	[− 1.011, − 0.283]	− 0.433(0.175)	− 2.469	0.014*	[− 0.776,− 0.089]
Variance of model					− 0.365(0.071)	− 5.146	0.000***	[− 0.503, − 0.226]	− 0.584(0.138)	− 4.231	0.000***	[− 0.854, − 0.313]
Intercept	0.422(0.103)	4.079	0.000***	[0.213,0.631]	0.402(0.092)	4.356	0.000***	[0.221,0.5823]	0.460(0.157)	2.930	0.003**	[0.152,0.767]
IC1	− 0.329(0.09)	− 3.438	0.001 **	[− 0.523, − 0.135]	− 0.428(0.201)	− 2.126	0.034*	[-0.826,-0.033]	− 0.423(0.149)	− 2.847	0.004**	[− 0.715,0.132]
Variance of model					− 0.397(0.071)	− 5.624	0.000***	[− 0.823, − 0.033]	− 0.768(0.138)	− 5.563	0.000***	[− 1.039, − 0.498]

Significance codes: ‘***’ ≤ 0.001; ‘**’ ≤ 0.01; ‘*’ ≤ 0.05

## Discussion

We combined PSM and Tobit regression techniques to investigate the impact of IC adoption on hospital efficiency calculated through DEA methods after controlling for potential confounding. We demonstrated that the adoption of IC had a positive effect on hospital efficiency after controlling for a range of covariates.

It is found that the mean efficiency score of all the sampled hospitals under the VRS assumption was 0.81, but it fell to 0.76 when the CRS was used. This may be explained by that hospitals’ size is assumed to be not relevant to their efficiency under the CRS assumption, but large hospitals were assumed to achieve a higher level of efficiency than small hospitals under the VRS assumption [44]. Our results also suggest that the type of IC had a differential effect on hospital efficiency with vertical and administrative integration models yielding higher efficiency scores compared to the contractual integration model. Given the degree of governmental control over institutions in China [46], it was anticipated that the administrative model of IC would fare better in terms of hospital efficiency than the contractual model. At the same time, the success of the vertical integration model may be attributed to the rapid development of both information technology and artificial intelligence, which offers the potential to enhance outcomes and conserve resource inputs [56].

The main study finding that IC hospitals were more efficient than non-IC hospitals is congruent with previous research in the literature [25–29]. However, our study is at variance with literature that reported negative effects of integration on efficiency [30]. This discrepancy could be explained by differences in the unit of analysis and the way integration was measured in previous studies. Integration in those studies was measured by the number of integrated HIV and sexual and reproductive health services in the same clinical room. This may reveal that although integration might improve hospital efficiency in general, there might be negative effects of integration per clinical room.

Our study explored the pathways through which IC might promote hospital efficiency. Our research demonstrates that IC was statistically associated with a range of input and output variables, which may reveal the pathways through which IC impacts hospital efficiency. This is consistent with a previous research that has shown that IC could improve health services utilization significantly and therefore lead to higher efficiency [57]. What’s more, our study demonstrated specific relationships between IC and each input/output variable. It was found that IC could influence a set of hospital output variables, such as length of stay, inpatient visits, emergency visits and the number of patients discharged. Meanwhile, IC was also found to be associated to a range of input variables, including number of physicians, nurses, other employees, and hospital beds. These findings provide preliminary evidence about how IC changes hospital efficiency by reallocating medical resources and impacting hospital production processes.

Our research has important policy implications which may be helpful for future healthcare reforms. This research showed how the adoption of IC resulted in improvements to hospital efficiency. Opportunities to foster the development of those types of IC that have the greatest potential to enhance hospital efficiency may be pursued. Policies such as “Guiding Opinions on Promoting the Integration of Healthcare and Elderly Care Services” [58] would help the diffusion of such IC models across China. Moreover, there is the potential to expand the scope of IC beyond hospitals to other health care settings.

Our research has some strengths: First, to the best of our knowledge, this is the first paper to investigate the influence of IC on hospital efficiency in China. This research adds empirical evidence to the pool of global IC evaluative research and offers practical suggestions for IC reform. Moreover, PSM was used in our study to remove potential confounding associated with the uptake of IC and Tobit regression analysis was adopted to deal with the censoring of the dependent variable (in our case hospital inefficiency). These techniques help to ensure reliable and robust estimates. Third, our research included all hospitals in one Chinese city and therefore was representative of hospitals in that city.

Several limitations warrant recognition: First, we were unable to assess the role of environmental factors, such as population size and poverty, on hospital efficiency due to a lack of available data. Future studies with datasets across different administrative regions will allow for more precise conclusions. However, our research results are still robust in terms of controlling the covariates included by our research. Second, there was an absence of cross-sectional data to explain the long-term causal effects of IC on hospital efficiency. Nevertheless, our research results were still useful in the evaluation of associations and the short-term effects of IC on hospital efficiency. Third, we only have data on all hospitals in one city which limits the generalizability of our results. While this limitation is common in studies, we were fortunate to have the universe of hospitals in our study city included, and moreover, this study city is located in central China and is representative of all China in having average economic and social development. Consequently, our findings are still applicable to the role of IC on hospital efficiency in China. Fourth, while our study addressed a range of statistical concerns, we were still unable to resolve the potential for endogeneity of the relationship between IC and efficiency. A higher degree of integration can improve hospital efficiency, but an efficient hospital is also good at integrating health services [15]. Such endogeneity problems could be addressed by applying appropriate instrumental variables in future studies.

## Conclusions

This study has demonstrated the potential gains to hospital efficiency in China associated with the adoption of IC. This study has also found that IC may enhance hospital efficiency through exerting impact on number of physicians, nurses, other staff, hospital beds, patients discharged, inpatient visits, emergency visits, and length of stay. The work has also highlighted the greater potential for gains in efficiency associated with the virtual and administrative models of IC relative to other types of IC. These findings may assist policy decision makers that are confronted with increased pressure on the health system due to an aging population and one with an increasing prevalence of chronic conditions. Integrated care has been shown to enhance health system performance and opportunities to facilitate uptake and remove barriers to its adoption have potential to improve population health and conserve scare health care resources.

## Supplementary Information

**Additional file 1**: **Table S1**. Descriptive statistics for matched sample (mean). **Table S2**. Balance diagnostics of matched sample (SD). **Figure S1**. Common support. **Figure S2**. Balance diagnostics of matched sample (mean). **Figure S3**. Balance diagnostics of matched sample (SD)

## Data Availability

The datasets generated and/or analysed during the current study are not publicly available due to that the dataset involves private information about each hospital but are available from the corresponding author on reasonable request.

## Abbreviations

IC: Integrated care
DEA: Data Envelopment Analysis
NCD: Non-communicable disease
CRS: Constant returns to scale
VRS: Variable returns to scale
PSM: Propensity score matching

## References

[1] National Bureau of Statistics of China. China statistical yearbook. Beijing: National Bureau of Statistics of China; 2016. http://www.stats.gov.cn/tjsj/ndsj/2016/indexch.htm. Accessed 30 Jun 2020.

[2] Leeder S, Raymond S, Greenberg H, Liu H, Esson K (2005). A race against time: the challenge of cardiovascular disease in developing economies.

[3] National Health Commission of the People’s Republic of China. China health statistical yearbook; 2016.

[4] Yong Z, Yichun T, Xuchang X (2018). Blue book of smart elderly care: report on development of China smart elderly care industry 2018.

[5] Gobbens RJJ, Luijkx KG, Wijnen-Sponselee MTH, Schols JMGA (2010). Towards a conceptual definition of frail community-dwelling older people. Nurs Outlook.

[6] Kodner D (2009). All together now: a conceptual exploration of integrated care. Healthcare Q.

[7] Fan W, Yanfei G, Paul K, Jiang Y, Yu M, Li X, Zheng Y, Xu J (2013). Prevalence of major chronic conditions among older Chinese adults: the Study on Global AGEing and adult health (SAGE) Wave 1. PLoS ONE.

[8] Kodner D, Spreeuwenberg C (2002). Integrated care: meaning, logic, applications, and implications-a discussion paper. Int J Integr Care.

[9] Leutz W (1999). Five laws for integrating medical and social services: lessons from the united states and the United Kingdom. Milbank Q.

[10] World Health Organization. Integrated health services—what and why? Making health systems work. Geneva: World Health Organization; 2008. https://www.who.int/healthsystems/technical_brief_final.pdf. Accessed 30 Jun 2020.

[11] Pourmohammadi K, Hatam N, Shojaei P, Bastani P (2018). A comprehensive map of the evidence on the performance evaluation indicators of public hospitals: a scoping study and best fit framework synthesis. Cost Eff Resour Alloc.

[12] Shortell S (2000). Remaking health care in America: the evolution of organized delivery systems.

[13] Ahmed S, Hasan MZ, Laokri S (2019). Technical efficiency of public district hospitals in Bangladesh: a data envelopment analysis. Cost Eff Resour Alloc.

[14] Spoorenberg SLW (2017). Integrated care for older adults improves perceived quality of care: results of a randomized controlled trial of embrace. J Gen Intern Med.

[15] Looman WM, Fabbricotti IN, Kuyper RD, Huijsman R (2016). The effects of a pro-active integrated care intervention for frail community-dwelling older people: a quasi-experimental study with the GP-practice as single entry point. BMC Geriatr.

[16] Pollina LD, Guessous I, Petoud V, Combescure C, Buchs B, Schaller P, Kossovsky M, Gaspoz JM (2017). Integrated care at home reduces unnecessary hospitalizations of community-dwelling frail older adults: a prospective controlled trial. BMC Geriatr.

[17] Damery S, Flanagan S, Combes G (2016). Does integrated care reduce hospital activity for patients with chronic diseases? An umbrella review of systematic reviews. BMJ Open.

[18] Theodoridou A, Hengartner MP, Gairing SK, Jäger M, Ketteler D, Kawohl W, Lauber C, Rössler W (2015). Evaluation of a new person-centered integrated care model in psychiatry. Psychiatr Q.

[19] Crane HM, Fredericksen RJ, Church A, Harrington A, Ciechanowski P, Magnani J, Nasby K, Brown T, Dhanireddy S, Harrington RD, Lober WB, Simoni J, Safren SA, Edwards TC, Patrick DL, Saag MS, Crane PK, Kitahata MM (2016). A randomized controlled trial protocol to evaluate the effectiveness of an integrated care management approach to improve adherence among HIV-infected patients in routine clinical care: rationale and design. JMIR Res Protocols.

[20] Titova E, Salvesen Ø, Bentsen SB, Sunde S, Steinshamn S, Henriksen AH (2017). Does an integrated care intervention for COPD patients have long-term effects on quality of life and patient activation? A prospective, open, controlled single-center intervention study. PLoS ONE.

[21] Titova E, Steinshamn S, Indredavik B, Henriksen AH (2015). Long term effects of an integrated care intervention on hospital utilization in patients with severe COPD: a single centre controlled study. Respir Res.

[22] Uga A, Kulkarni S, Heeramun V, Bottum K (2017). Evaluation of a model of integrated care for patients with chronic medical and psychiatric illness. Psychosomatics.

[23] Berghöfer A, Hubmann S, Birker T, Hejnal T, Fischer F (2016). Evaluation of quality indicators of integrated care in a regional psychiatry budget-a pre-post comparison by secondary data analysis. Int J Integr Care.

[24] Ameh S, Gomez-Olive F, Kahn K, Tollman SM, Klipstein-Grobusch K (2017). Relationships between structure, process and outcome to assess quality of integrated chronic disease management in a rural south African setting: Applying a structural equation model. BMC Health Serv Res.

[25] Sekhri N, Feachem R, Ni A (2011). Public-private integrated partnerships demonstrate the potential to improve health care access, quality, and efficiency. Health Aff.

[26] Ozcan YA, Luke RD (2011). Health care delivery restructuring and productivity change: assessing the veterans integrated service networks (VISNs) using the malmquist approach. Med Care Res Rev.

[27] Pandey A, Gireesh A, Viner R (2019). Feasibility, acceptability, and effectiveness of young people-specific, integrated out-of-hospital services: a protocol for a systematic review. Syst Rev.

[28] Pomerantz A, Cole BH, Watts BV, Weeks WB (2008). Improving efficiency and access to mental health care: combining integrated care and advanced access. Gen Hosp Psychiatry.

[29] Wan TTH, Lin BY, Ma A (2002). Integration mechanisms and hospital efficiency in integrated health care delivery systems. J Med Syst.

[30] Obure CD, Jacobs R, Guinness L, Mayhew S, Mayhew S, Vassall A (2016). Does integration of HIV and sexual and reproductive health services improve technical efficiency in Kenya and Swaziland? An application of a two-stage semi parametric approach incorporating quality measures. Soc Sci Med.

[31] Machta RM, Maurer KA, Jones DJ, Furukawa MF, Rich EC (2019). A systematic review of vertical integration and quality of care, efficiency, and patient-centered outcomes. Health Care Manage Rev.

[32] Health Commission of the People’s Republic of China, Ministry of Civil Affairs of the People’s Republic of China. Notice Regarding the Determination of the First Batch of National-level Pilot Cities of Integrated Care (2016) No.644. Beijing: Health Commission of the People’s Republic of China and Ministry of Civil Affairs of the People’s Republic of China; 2016.

[33] Amy TH (2016). An investigation of approaches to performance measurement: applications to long-term care in Ontario.

[34] Atilgan E, Çalışkan Z (2015). The cost efficiency of Turkish hospitals: a stochastic frontier analysis. İktisat İşletme ve Finans.

[35] Atilgan E (2016). Stochastic frontier analysis of hospital efficiency: does the model specification matter. J Bus Econ Financ.

[36] Atilgan E (2016). The Technical efficiency of hospital inpatient care services: an application for Turkish public hospitals. Bus Econ Res J.

[37] Scott KW, Orav EJ, Cutler DM, Jha AK (2017). Changes in hospital–physician affiliations in U.S. hospitals and their effect on quality of care. Ann Intern Med.

[38] Koch TG, Wendling BW, Wilson NE (2017). How vertical integration affects the quantity and cost of care for Medicare beneficiaries. J Health Econ.

[39] Otaya I, Oztaysi B, Onar SZ, Kahramanb C (2017). Multi-expert performance evaluation of healthcare institutions using an integrated intuitionistic fuzzy AHP&DEA methodology. Knowl-Based Syst.

[40] Acton QA. Stroke: New insights for the healthcare professional. Atlanta: ScholarlyEditions; 2013.

[41] Cheng Z, Tao H, Cai M, Lin H, Lin X, Shu Q, Zhang R (2015). Technical efficiency and productivity of Chinese county hospitals: An exploratory study in Henan province. China BMJ Open.

[42] Worthington A (2004). Frontier efficiency measurement in health care: a review of empirical techniques and selected applications. Med Care Res Rev.

[43] Kumbhakar SC, Heshmati A (1996). DEA, DFA and SFA: A comparison. J Prod Anal.

[44] Ngobeni V, Breitenbach MC, Aye GC (2020). Technical efficiency of provincial public healthcare in South Africa. Cost Eff Resour Alloc.

[45] Ning Li, Jian W (2008). The technical efficiency analysis on China’s public hospitals: application of data envelopment analysis. Chin J Health Policy.

[46] Zhu L, Peng ZX, Liu LH (2019). Combining resource, structure and institutional environment: a configurational approach to the mode selection of the integrated healthcare in county. Int J Environ Res Public Health.

[47] Staffa SJ, David Z (2018). Five steps to successfully implement and evaluate propensity score matching in clinical research studies. Anesth Analg.

[48] Garrido MM, Kelley AC, Paris J, Roza K, Meier DE, Morrison RS, Aldridge MD (2014). Methods for constructing and assessing propensity scores. Health Res Educ Trust.

[49] Caliendo M, Kopeinig S (2008). Some practical guidance for the implementation of propensity score matching. J Econ Surv.

[50] Austin PC (2011). An introduction to propensity score methods for reducing the effects of confounding in observational studies. Multivariate Behav Res.

[51] Rosenbaum PR, Rubin DB (1985). Constructing a control group using multivariate matched sampling methods that incorporate the propensity score. Am Stat.

[52] Stuart WA, Lee BK, Leacy FP (2013). Prognostic score-based balance measures can be a useful diagnostic for propensity score methods in comparative effectiveness research. J Clin Epidemiol.

[53] Rubin DB (2001). Using propensity scores to help design observational studies: application to the tobacco litigation. Health Serv Outcomes Res Method.

[54] Austin PC (2009). Balance diagnostics for comparing the distribution of baseline covariates between treatment groups in propensity-score matched samples. Stat Med.

[55] R Core Team. R: A language and environment for statistical computing. Vienna: R Foundation for Statistical Computing; 2013.

[56] Tian MM, Xu XD, Zhu K (2014). Situation and effect of vertical integrated of rural health services: a case study in Dafeng County of Jiangsu Province. Chin J Health Policy.

[57] Solberg BCJ, Dirksen CD, Nieman FHM (2014). Introducing an integrated intermediate care unit improves ICU utilization: a prospective intervention study. BMC Anesthesiol.

[58] National Health Commission of the People’s Republic of China, Ministry of Civil Affair of the People’s Republic of China, National Development and Reform Commission, et al. Guiding opinions on promoting the integration of healthcare and elderly care services. Beijing: National Health Commission of the People’s Republic of China, Ministry of Civil Affair of the People’s Republic of China, National Development and Reform Commission, et al. 2015. http://www.gov.cn/zhengce/content/2015-11/20/content_10328.htm. Accessed 20 Feb 2019.

