# Supplementary material for: Can integrated care improve the efficiency of hospitals? Research based on 200 Hospitals in China
Source: Cost Eff Resour Alloc. 2021 Sep 22;19:61. doi: 10.1186/s12962-021-00314-3 (PMC8456592; doi:10.1186/s12962-021-00314-3)
Supplement: Supplementary file 1 — Additional file 1: Table S1. Descriptive statistics for matched sample (mean). Table S2. Balance diagnostics of matched sample (SD). Figure S1. Common support. Figure S2. Balance diagnostics of matched sample (mean). Figure S3. Balance diagnostics of matched sample (SD) [file 12962_2021_314_MOESM1_ESM.docx]

**Additional file:**

**Table S1.** Descriptive statistics for matched sample (mean)

|  | ***Before-match*** | | | ***After-match*** | | |
| --- | --- | --- | --- | --- | --- | --- |
| ***Variable***  ***(Mean(SD))*** | ***Non-IC***  ***(N=176)*** | ***IC***  ***(N=24)*** | ***P-value*** | ***Non-IC***  ***(N=23)*** | ***IC***  ***(N=23)*** | ***P-value*** |
| WHC=0 | 140 (79.5) | 13 (54.2) | 0.013 | 15 (65.2) | 13 (56.5) | 0.763 |
| WHC=1 | 36 (20.5) | 11 (45.8) |  | 8 (34.8) | 10 (43.5) |  |
| RMA | 0.00 (0.01) | 0.01 (0.02) | <0.001 | 0.01 (0.01) | 0.01 (0.02) | 0.746 |
| ROPA | 10.40 (12.20) | 23.83 (43.44) | 0.001 | 18.17 (27.63) | 24.45 (44.31) | 0.567 |
| NAPP | 5.05 (5.10) | 5.41 (6.80) | 0.756 | 4.59 (7.82) | 5.54 (6.93) | 0.665 |
| NAPN | 3.80 (3.99) | 2.84 (3.01) | 0.258 | 2.48 (2.54) | 2.91 (3.06) | 0.604 |
| TNS | 1.52 (2.64) | 6.00 (8.06) | <0.001 | 4.35 (5.64) | 4.91 (6.19) | 0.749 |

**Table S2.** Balance diagnostics of matched sample (SD)

|  | ***Before (%)*** | ***After (%)*** | ***Reduction (%)*** |
| --- | --- | --- | --- |
| RMA | 49.803 | 9.622 | 80.680 |
| WHC | 55.986 | 17.889 | 68.047 |
| TNS | 74.683 | 9.501 | 87.278 |
| NAPP | 6.005 | 12.862 | -114.188 |
| NAPN | 27.167 | 15.397 | 43.325 |
| ROPA | 42.081 | 17.009 | 59.580 |
| Mean | 42.621 | 13.713 | 37.454 |

The reduction of the standardized difference equals to the ratio of (absolute

standardized difference before matching - absolute standardized difference after

matching) to absolute standardized difference before matching.

**Figure S1.** Common support

**
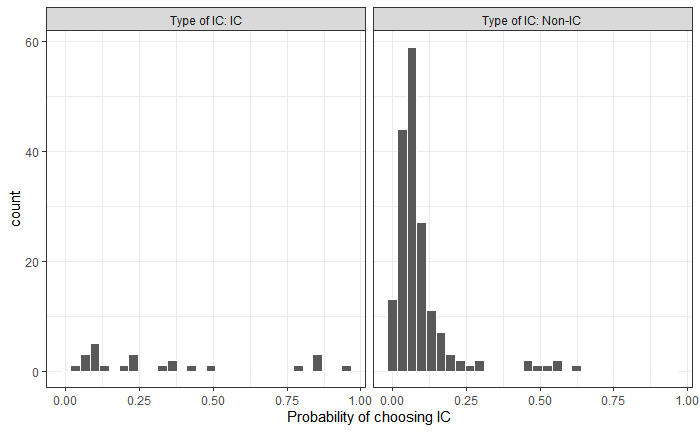
**

**Figure S2.** Balance diagnostics of matched sample (mean)

**
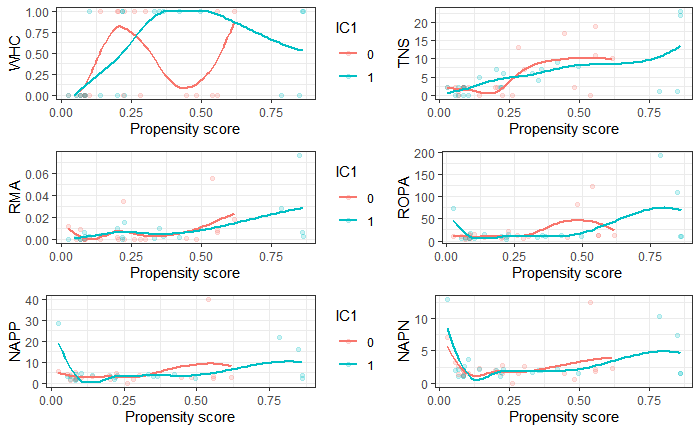
**

**Figure S3.** Balance diagnostics of matched sample (SD)

**
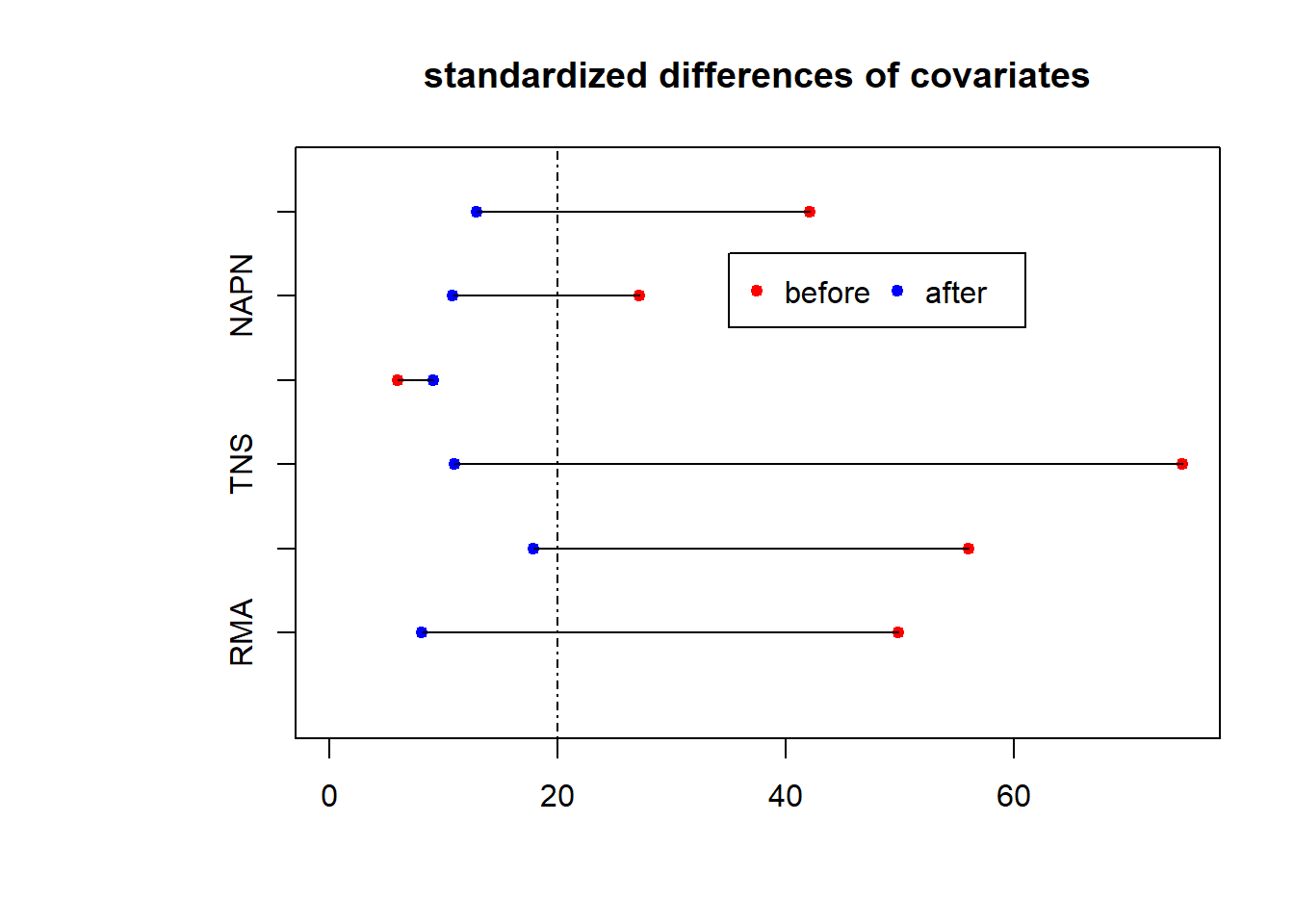
**
